# Saliency Detection Using Sparse and Nonlinear Feature Representation

**DOI:** 10.1155/2014/137349

**Published:** 2014-05-08

**Authors:** Shahzad Anwar, Qingjie Zhao, Muhammad Farhan Manzoor, Saqib Ishaq Khan

**Affiliations:** ^1^Beijing Key Laboratory of Intelligent Information Technology, School of Computer Science, Beijing Institute of Technology, Beijing 100081, China; ^2^School of Automation, Beijing Institute of Technology, Beijing 100081, China; ^3^Centres of Excellence in Science and Applied Technologies, Islamabad 44000, Pakistan

## Abstract

An important aspect of visual saliency detection is how features that form an input image are represented. A popular theory supports sparse feature representation, an image being represented with a basis dictionary having sparse weighting coefficient. Another method uses a nonlinear combination of image features for representation. In our work, we combine the two methods and propose a scheme that takes advantage of both sparse and nonlinear feature representation. To this end, we use independent component analysis (ICA) and covariant matrices, respectively. To compute saliency, we use a biologically plausible center surround difference (CSD) mechanism. Our sparse features are adaptive in nature; the ICA basis function are learnt at every image representation, rather than being fixed. We show that Adaptive Sparse Features when used with a CSD mechanism yield better results compared to fixed sparse representations. We also show that covariant matrices consisting of nonlinear integration of color information alone are sufficient to efficiently estimate saliency from an image. The proposed dual representation scheme is then evaluated against human eye fixation prediction, response to psychological patterns, and salient object detection on well-known datasets. We conclude that having two forms of representation compliments one another and results in better saliency detection.

## 1. Introduction

Vision is the primary source of information that the human brain uses to understand the environment it operates in. The eyes capture light which results in information in the order of 10^9^ bits every second. In order to efficiently process such huge amount of information, the brain uses visual attention to seek out only the most salient regions in the visual field. Designing an artificial system, the designer endeavors it to be maximally efficiency; real-time and computational frugal, like biological systems. Thus biologically inspired concepts are regularly used in designing various computational algorithms. In computer vision, a number of computational algorithms are designed based on visual attention in primates. Such visual saliency models have shown reasonable performance and are used in many applications like robot localization [1], salient object detection [2], object tracking [3], video compression [4], thumbnail generation [5], and so forth. A detailed discussion on the subject can be found in [6].

There are two distinct neural pathways underlying visual attention in the primate brain. The top-down [6], goal driven mechanism is slow and based on learning, experience, and recall. On the other hand, the sensor driven bottom-up [6] pathway is fast and deals only with the presented stimulus. Many computer vision algorithms utilize a bottom-up approach to find salient features in a set of images presented to it. Using such an approach would require efficient encoding of various image variables that may represent features in the image.

How an image is being represented for saliency detection is very important. The first computational model for saliency by Itti et al. [7] used color, orientation, and intensity to represent an image. These features are inspired by the feature integration theory (FIT) [8]. Other stimulus properties that drive visual attention could be motion, occlusion like optical flow [9], skin hue [10], texture contrast [11], wavelet [12], face [13], and gist [14, 15]. A summary of various features used in saliency computation can be found in [6]. Here, we limit our discussion to sparse [16] and nonlinear representations [17].

*Adaptive Sparse Representation.* A simple cell in visual cortex is characterized by its location within the field of view, its spatial frequency selection, and its orientation. It is believed that the visual cortex is being evolved in such a way that it could efficiently process natural images, the kind of visual stimuli it would experience in natural conditions. Learning statistics of natural images could lead to development of simple cell like receptive fields. Thus a number of studies [18, 19] have used learning methods along with natural stimuli to this end. For example, Olshausen and Field [20] have shown that, assuming sparseness the basis functions learnt from an ensemble of natural images can fulfill the properties of a simple cell's receptive field.

Sparse representation means a representation of the data such as the constituent components are rarely active. For such a representation, a dictionary of basis is learnt from an ensemble of natural image patches with a condition that the respective weights of the basis coefficients are sparse and rarely active and most of the time have zero value. Sparse representation is also an efficient way of processing images for various applications like classification, face recognition, image denoising, and saliency computation [16].

An important observation about human visual systems is its adaption to a new environment. Several studies [21] have shown an adaptive behavior of neurons in visual cortex. Based on such observations, a saliency model utilizing an adaptive sparse representation has been proposed [22]. The basis or the dictionary for an adaptive sparse representation does not remain fixed but changes with every stimulus and thus better represents the current environment. AWS [23] also used an adaptation but by whitening the features as per particular image structure.

Independent component analysis (ICA) is a very popular technique in computer vision for multilinear data analysis. ICA gives basis functions which are statistically independent as well as non-Gaussian. The aim of an ICA algorithm is to recover independent basis from their observed linear mixture. An image is represented using an ICA basis functions, then the coefficients of these basis are sparse, similar to neural receptive fields in the visual cortex [20].

In our two pronged approach, one deals with an adaptive, sparse representation of natural images. We approach this part using ICA basis functions learnt from individual images. The resulting dictionary changes as images are introduced and are hence considered to be adaptive in nature. This representation is similar to that in [22] where a global approach [6] based on Shannon theory [24] is used to estimate saliency. Our contribution to this end is the use of a different biologically plausible mechanism to estimate saliency in an adaptive sparse representation. We later show that an accurate representation of the stimulus leads to a reasonably good accuracy in computing the center surround difference (CSD). 

*Nonlinear Representation.* In visual saliency estimation usually, the feature representation takes the form of a linear transformation of the original data and various popular transformations like ICA, factor analysis, projection pursuit, or principal component analysis (PCA) are used for this purpose. There are some biological evidences which support the use of nonlinear feature representations. In [25], the authors showed that the invariance property in V1 can be result of a nonlinear operation on features. The literature on nonlinear representation of features for the saliency modeling is very limited. The properties of nonlinear representation depend on the nonlinear kernel and the features input to that kernel. In [26] local steerable kernels, LSK, with only gradient information are used for such a representation. A recent approach [17] shows that all features are combined in matrix form and then a covariance based integrated nonlinear representation gives good results. These nonlinear representations claim to better capture the data structure than a linear representation and integrate all features to give a single unified representation, as used by both [17, 26].

Inspired by the concept in [17], we choose covariance based feature representation but we do not combine all features and channels to form integrated covariance matrices. Instead, we rather modify the approach and show that using only color with spatial information, integrated in a nonlinear covariance representation, performs better than all features integrated nonlinearly [17]. Here also CSD is utilized to compute saliency. In Section 5, we will give a comparison to show that the proposed representation gives better eye-fixation predictions. Also, the use of only color information and how it is complemented by the sparse representation will be explained there.

**Figure 1:**
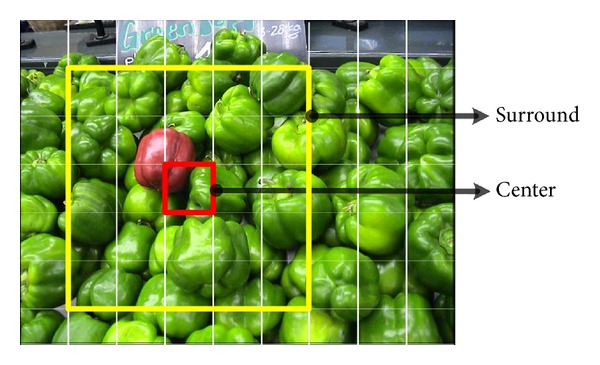
A depiction of center surround difference, where the patch in consideration is in red rectangle and the patches in yellow rectangular region are surrounding area from which the dissimilarity is checked.

There is a body of literature [27] on biological plausibility of CSD mechanism. CSD means that a stimulus area is conspicuous if it is different than immediate surroundings. This mechanism is utilized for saliency estimation in many forms; Itti et al. [7] used difference-of-Gaussian (DoG) filter, Gao [28] utilized KL-divergence based on histograms, and Borji and Itti [29] used average weighted patch dissimilarity. For our model, we also dwell on average weighted patch dissimilarity [22, 29, 30]. It is explained in Figure 1, the red window is the stimulus patch under consideration, and in order to compute CSD, we calculate the difference (norm) with all the neighboring patches (yellow window) followed by weighting and averaging the difference values. Here weights depend upon the distance between centers of two patches. Same procedure is then repeated for all the patches in the image and thus CSD assigns higher value to patches which are significantly different than the surroundings.

To summarize, in this paper, restricting ourselves only to bottom up visual saliency, our aim is to predict human eye fixation with a saliency model that utilizes dual image representation. We use both an adaptive sparse and a covariance based approach for image feature representation. A center surround difference (CSD) approach is used on both representations to compute saliency maps. To the best of our knowledge, this is the first time that a CSD mechanism is used with an adaptive sparse image representation. Moreover, the proposed scheme of using only color information in non-linear form remarkably improve results. Both saliency maps are fused at a later stage to form a net saliency map, which represents salient features better than saliency maps from the two independent representations.

This paper is organized as follows. Related work is given in Section 2.  Section 3 covers proposed model and the mathematical formulation of both representations and saliency computation.  Section 4 covers experimentation and results section. A detailed discussion about the contribution in both representations along with certain necessary comparisons is given in Section 5 followed by conclusion in Section 6.

## 2. Related Work

Initial visual saliency models take inspiration from feature integration theory (FIT) [8] and guided search models [31]. The first practical implementation of a saliency model based on these theories is presented by Itti et al. [7], where a number of contrast features are learnt in parallel and fused together to make a topographic map. Later Le Meur et al. [32] presented another cognitive model based on a contrast sensitivity function along with the incorporation of a CSD mechanism and perceptional decomposition. There are numerous other models proposed, which utilize different bioinspired features and mechanisms for saliency detection like GIST [33], PCA [30], ICA [16], histogram of local orientations, symmetry [34], depth, entropy [16], texture, and motion [35].

Apart from cognitive models, different probability based models are also presented. These models incorporate image statistics and learn probability distributions using current image or ensemble of images for saliency estimation. Itti and Baldi [36] defined a Bayesian surprise, using Kullback-Leibler (KL) distance for posterior and prior beliefs. Torralba et al. [33] used contextual evidence to further consolidate low level saliency. Harel et al. [37] approached the saliency problem using the probabilistic graphical models. Hou and Zhang [38] used a global approach based on Fourier transform to find saliency. He proposed that the residue of original and smoothed amplitude spectrum of a Fourier transform contains information about salient region in an image. Later, it was shown [4] that the phase of a Fourier transform contains the essential location information rather than the amplitude. There are several other models which use Fourier transform and are classified as frequency based models [2]. In these models, respective color channels are individually processed for saliency detection and finally all maps are fused in a single saliency map. In contrast a quaternion approach is proposed [39] to use a unified frame work to process all color channels. The quaternion framework also allows incorporation of a fourth channel like motion in a very elegant manner. Some models use learning techniques that incorporate human eye fixation in their saliency models. Kienzle et al. [40] used Human eye fixations for deriving a learned model while Tilke et al. [41] trained support vector machines (SVM) on image patches with low, intermediate, and high level features to compute saliency. Apart from above-mentioned approaches, different new techniques like redundancy, rectangular window, SNR, and regression have shown remarkable results in saliency modeling. In a different approach, Wang et al. [42] proposed site rate entropy for computing saliency using framework of graphical models.

Some approaches use information theoretic frameworks to model saliency like Bruce and Tsotsos [16] gives the idea of information maximization (AIM). Using a biological structure [20] of sparse representation and Shannon's [24] formulation of self-information, inverse probability of a patch in the entire image, Bruce and Tsotsos [16] computed saliency. This self-information can be considered as a global measure of saliency. There are various extensions of using sparse representations of images using a learned dictionary for saliency computation. Recently, Sun et al. [22] proposed that since biological systems are adaptive, an adaptive dictionary is more representative and thus he used principle of self-information for saliency computation using adaptive basis. AWS [23] also used adaption and it works on the principle that a statistical distance in a representative space gives saliency. This representative space is computed by whitening the basis to the structure of a particular image. This scheme uses multiscale and multistage operations on features and uses an efficient way to overcome the computation complexity in whitening.

In [29], Borji and Itti proposed that local and global measures are complementary and used both center surround and self-information for saliency computation. Moreover they showed that multiple color spaces are useful in better saliency estimation. There are some saliency models [17, 26] which rely on nonlinear representation of features and on the integration of various features and channels. In [26], gradient features are used in a nonlinear representation based on local steerable kernels (LSK) for image representation, while the author in [17] proposes a nonlinear integration using covariance matrices. This paper also incorporates first order image statistics in covariance matrices to better estimate saliency. Moreover [17, 26] also solve various features and respective channels integration issue by putting forth a single unified form.

Our saliency model is inspired from mainly two types of models, sparse [16, 20] and nonlinear representation [27]. We propose a novel dual approach based on both sparse and nonlinear feature representation. Inspired by biological evidence of neural receptive field properties [20] that efficiently process natural images in a sparse manner, we use a sparse image representation. Moreover, in order to represent adaptivity of neurons to better tackle a new environment, we use an adaptive basis dictionary in an ICA approach [22]. Thus our proposed method simultaneously uses sparsity and adaptivity. In literature, the model similar to our adaptive sparse representation is [22]. In [22] Sun et al. used an information theoretic global approach for saliency computation but we use a more bioplausible local CSD for saliency computation. Secondly, we propose nonlinearly integrated representation of single feature channels, color along with spatial information for saliency computation. Our approach is a modification of the model proposed in [17], where all features and channels are nonlinearly integrated using covariance matrices, although we propose that only color information is enough and it can better estimate saliency in our framework. Here also a CSD approach is used for saliency computation. Finally a combined saliency map is formed by fusing the outputs given by the two representations. 

*Contributions.* Major contributions of this work can be summarized as follows.

1. A novel dual image feature representation: simultaneous sparse and nonlinear feature representation.
2. CSD based saliency computation in adaptive sparse representation.
3. Only color based nonlinear integrated covariance representation followed by CSD computation.
4. Improved results with comparison to other states of the models that are established by extensive testing on popular eye fixation prediction datasets and on a salient object detection dataset.

## 3. Proposed Model

**Figure 2:**
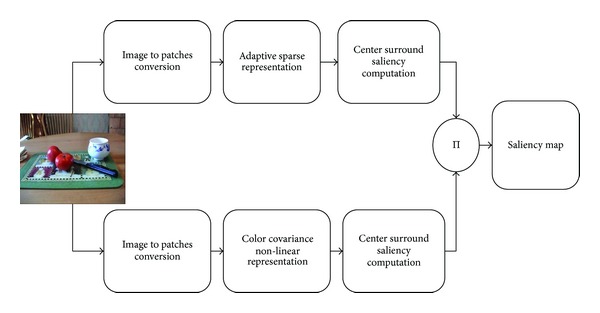
Proposed model for saliency computation.

Our proposed scheme is given in Figure 2. An input image is being simultaneously represented in sparse and nonlinear form. Then saliency is computed by local center surround operation and finally both maps are combined to form a single saliency map.

For sparse representation, we break an image into patches and perform independent component analysis to derive basis. Later these basis with sparse coefficient are used to represent the image. Furthermore, again after converting image into patches, we only take color information and integrate all channels in a nonlinear fashion, using covariance matrices along with spatial information, to represent an image patch.

### 3.1. Mathematical Modeling

In this section, we cover mathematical formulation of sparse representation, nonlinear representation, and saliency computation. Some necessary discussion is included to elaborate few concepts and also some references will be given to avoid unnecessary formulation of the well-known concepts.

*Sparse Representation with ICA.* We use an ICA based image representation; thus, an input image, *I*, can be sparsely represented as

(1) $$\begin{array}{c} I^{\prime }=As, \end{array}$$

where *A* is a dictionary consisting of a set of basis and **s** consists of respective coefficients. In our case, we learn *A* from every input image; thus, we adapt dictionary for every input stimulus. This approach results in minimum information loss which is a basic drawback of a fixed dictionary, learned from an ensemble of images. The sparse coefficients are learned by projecting an input image to the basis such as

(2) $$\begin{array}{c} s=WI, \end{array}$$

where

(3) $$\begin{array}{c} W=A^{-1}. \end{array}$$

**Figure 3:**
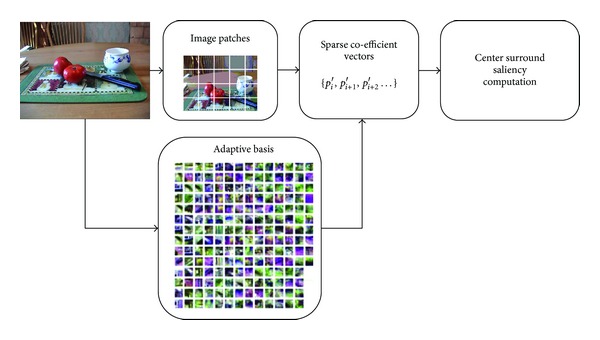
Saliency computation in adaptive sparse representation.

The basis have dimensions the same as the patches formed from the input image. Finally, *I*′ in patches form can be represented as

(4) $$\begin{array}{c} I^{\prime }=\overset{n^{\prime }}{\underset{k=1}{⨊}}{Ap}_{k}^{\prime }, \end{array}$$

where *p*_*k*_′ is the *k*th patch's sparse coefficient vector consisting of *m*′ co-efficients and there are total *n*′ number of patches in *I*′. Moreover ⨊ represents a function that reshape and arranges patches at respective position to form an image.  Figure 3 gives the depiction of the whole process.

*Nonlinear Representation with Covariance Matrices.* Our feature matrix, *F*, is based on raw RGB color space values of *I* along with pixel position information,

(5) $$\begin{array}{c} F=\left[I_{R},I_{G},I_{B},x,y\right], \end{array}$$

where every pixel in *F* is a 5-dimensional vector, {*f*_*i*_}_*i*=1,2,…,*p*_, where *p* is the total number of pixels in the image. Our features are different than those used in E. Erdem and A. Erdem [17] since we do not incorporate any gradient information in our feature matrix, *F*. In (5), color along with spatial information is used rather than E. Erdem and A. Erdem [17] approach of making a features matrix consisting of all features.

**Figure 4:**
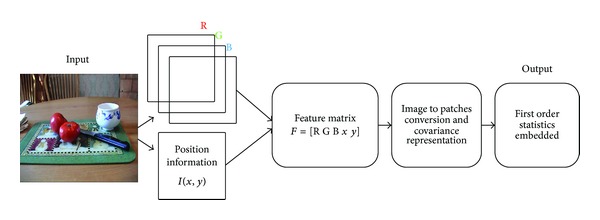
Nonlinear representation of an input image.

The next step is nonlinear representation of *F* using covariance matrices along with first order statistics [17]. Tuzel et al. [43] gave the concept of encoding a patch by a covariance matrix. Later it was used in many applications. In saliency domain, E. Erdem and A. Erdem [17] used patch covariance with first order statistics for image feature representation and we will dwell on his approach for our case. Thus calculating local covariance matrices for an image patch *p*_*k*_, we get

(6) $$\begin{array}{c} C\left(p_{k}\right)=\frac{1}{m-1}\overset{m\;}{\underset{i=1}{\sum }}\left(f_{i}-\mu_{k}\right){\left(f_{i}-\mu_{k}\right)}^{T}, \end{array}$$

where a patch *p*_*k*_ consists of *m* pixels with mean *μ*_*k*_. Now first order statistics is incorporated in the covariance matrices using the method mentioned in [17]. Then the new representation of covariance matrices, with first order statistics embedded, for a patch is given by

(7) $$\begin{array}{c} \psi_{k}=\psi \left(C\left(p_{k}\right)\right), \end{array}$$

where function *ψ* embeds first order statistics in an input matrix. The final nonlinear feature representation of image with *ψ*_*k*_ represents a *k*th patch and *n* being total number of patches is given by

(8) $$\begin{array}{c} I^{\prime \prime }=\overset{n}{\underset{k=1}{⨊}}\psi_{k}, \end{array}$$

where also ⨊ function arranges patches at respective positions to form an image. The whole representation is given in Figure 4.

*Saliency Computation.* The saliency is computed by CSD operation and then extended to multiple scales. The CSD operation is shown in Figure 1, where a patch under consideration is in red rectangle, and surrounding area is highlighted in yellow rectangle. The saliency of red patch, *p*_*i*_, is given by its dissimilarity between *w* surrounding patches (yellow rectangle) as

(9) $$\begin{array}{c} S\left(p_{i}\right)=\frac{1}{w}\overset{w}{\underset{j=1}{\sum }}d\left(p_{i},p_{j}\right), \end{array}$$

where dissimilarity, *d*(*p*_*i*_, *p*_*j*_), between two patches is given by

(10) $$\begin{array}{c} d\left(p_{i},p_{j}\right)=\frac{||\alpha \left(p_{i}\right)-\alpha \left(p_{j}\right)||}{1+||X_{i}-X_{j}||}, \end{array}$$

where *X*_*i*_ and *X*_*j*_ are the central position of the patches *p*_*i*_ and *p*_*j*_. For the case of sparse representation, we have

(11) $$\begin{array}{c} \alpha \left(p_{i}\right)={\;p}_{i}^{\prime } \end{array}$$

and for nonlinear representation, we have

(12) $$\begin{array}{c} \alpha \left(p_{i}\right)=\psi_{i}=\psi \left(C\left(p_{i}\right)\right). \end{array}$$

Thus the saliency map for patch *p*_*i*_ derived from *I*′ and *I*′′ can be given as

(13) $$\begin{array}{c} S_{I^{\prime }}\left(p_{i}\right)=\frac{1}{w}\overset{w}{\underset{j=1}{\sum }}\frac{||p_{i}^{\prime }-{\;p}_{j}^{\prime }||}{1+||X_{i}-X_{j}||} \end{array}$$

and with *ψ*(*C*(*p*_*i*_)) being in vector form,

(14) $$\begin{array}{c} S_{I^{\prime \prime }}\left(p_{i}\right)=\frac{1}{w}\overset{w}{\underset{j=1}{\sum }}\frac{||\psi \left(C\left(p_{i}\right)\right)-\psi \left(C\left(p_{j}\right)\right)||}{1+||X_{i}-X_{j}||}. \end{array}$$

The multiscale saliency by sparse approach is given by

(15) $$\begin{array}{c} S_{I^{\prime }}\left(x\right)=N\left(\underset{l^{\prime }\epsilon L}{\prod }S_{I^{\prime }}^{l^{\prime }}\left(x\right)\right), \end{array}$$

and for nonlinear integrated approach is given by

(16) $$\begin{array}{c} S_{I^{\prime \prime }}\left(x\right)=N\left(\underset{l\epsilon L}{\prod }S_{I^{\prime \prime }}^{l}\left(x\right)\right), \end{array}$$

where *l*′ and *l* represent the number of scales and **N** shows normalization. Finally saliency map becomes

(17) $$\begin{array}{c} S_{I}=\;G_{\sigma }⊛\left(N\left(S_{I^{\prime }}*\;S_{I^{\prime \prime }}\right)\right), \end{array}$$

where *G*_*σ*_ represents the Gaussian smoothing by convolution, ⊛, operation and ∗ stands for multiplication operation.

## 4. Experimentation

In this section, we thoroughly evaluate the proposed model with three different experiments: human eye fixation prediction, salient-object detection, and response to various psychological patterns. The human eye fixation prediction is the basic and necessary test to check the performance of a saliency map against the collected eye fixation from several human subjects.

How well a saliency map distinguishes and highlights an object in an image shows its ability of salient object detection. The salient object detection capability of a model is evaluated by employing some metrics that compare the generated saliency map against the ground truth, made by manual labeling of the salient region in an image by human subjects. The psychological patterns give a qualitative analysis of the saliency model. These patterns are designed to check pop-up responses in different scenarios like orientation, conjunction and color, and so forth. Code (Matlab P-code) of the proposed model used for experimentation is available online [44].

### 4.1. Parameter Setting

Before pursuing the evaluation of the proposed model, we fix the parameters used to generate the saliency maps by our model. These parameters will remain the same for all the experiments. Derivation of these parameters will be discussed in the next section after the introduction of the datasets and the metric used for evaluation.

*Sparse Representation. *We resize all input images to 80 × 60 pixels and use only single scale, *l*′ = 1, for saliency computation. Patches of 5 × 5 pixels [22] are generated with sliding overlapping window from every input image to learn the basis for the dictionary and for the saliency computation. An ICA package, available online, FAST ICA [45], is used for this experimentation.

*Nonlinear Representation. *For nonlinear image representation, RGB color and position information is used in online available implementation of E. Erdem and A. Erdem [17]. The saliency is computed with the default parameters used in [17] that have every input image being resized to 512 × 512 pixels and five different patch sizes, *p*_*i*_ = {8,16,32,64,128} and thus *l* = 5, are used for saliency computation.

Finally normalized sparse representation's saliency map is rescaled to the size of nonlinear representation's saliency map and both maps are multiplied and normalized. Then the final saliency map is resized to the actual input image size and used for experimentation. The input image resolutions used in all the saliency algorithms for experimentation are given in Table 1.

### 4.2. Human Eye Fixation Prediction

In order to validate the proposed model with human eye fixation predictions, saliency maps are generated on three datasets and for a fair comparison shuffle area under the curve score(sAUC) is used to quantify the results.

*Dataset*. A reasonable dataset that can be used for evaluation of human eye fixation prediction must be complex and diverse enough so that performance can be thoroughly evaluated. In literature, Toronto [16] and Kootstra [34] datasets are the most popular and widely used datasets. IMSAL [2] is a relatively new dataset which we also used in our evaluation.

*Toronto* dataset was prepared by Bruce and Tsotsos [16] and it consists of 120 images each with 681 × 511 pixels. This dataset has both indoor and outdoor images. The eye fixation ground truth is based on 20 subjects who free viewed the images for few seconds.

*Kootstra* dataset was used in [34]. It consists of 101 images each with a resolution of 1024 × 768 pixels. These images consist of flowers, natural scenes, automans, and buildings. This dataset is significantly complex because it has many images with no explicit salient regions. The eye fixation ground truth available with this dataset is based on free viewing of 31 subjects for a few seconds.

*IMSAL *dataset is given by Li et al. [2]; it consists of 235 images, which are collected online through an internet search engine and some images were taken from literature. These images are divided into six categories, with 50 images having large salient regions, 80 images with intermediate salient regions, 60 images with small salient regions, 15 images with cluttered backgrounds, and 15 images with repeating distracters. These images give a good benchmark for performance evaluation because of the significant complexity given by variable size of salient objects, objects with clutter, and objects with distracters. The accompanied ground truth consists of both eye fixation information and binary masks created by human subjects, who manually marked the salient object in an image.

*Metric for Evaluation*. The most popular method to evaluate the performance of a saliency map is to calculate area under the curve (AUC) score of receiver operating characteristics (ROC) curve. At first, a saliency map is thresholded and used as a binary classifier with human eye fixations acting as positive set and some other points, uniformly random, as negative set to plot an ROC curve. The AUC of that ROC is calculated and used as a measure of performance comparison.

There are various variants of AUC available in literature and the basic difference between them is the choice of negative set points. We will use the shuffled area under the curve, sAUC, score because of its ability to cater for center bias [41]; since some models implicitly incorporate center bias which makes a fair comparison difficult to perform, it is becoming standard to present results with sAUC. In sAUC score, the positive points consists of human subjects eye fixation on that image and the negative set consists of all the fixation of subjects on the rest of the dataset images. The sAUC gives a 0.5 score on a center Gaussian blob, which is about the same as a random or chance score, whereas all the other versions of AUC [6] give very high score because they are affected by the center bias. For our experimentation, we used sAUC available online by Schauerte and Stiefelhagen [39]. We calculate every sAUC score for 20 times [47] and then use the mean value. We found that the standard deviation of the sAUC approximately ranges from 1*E* − 4 to 5*E* − 4 in our experiments.

**Figure 5:**
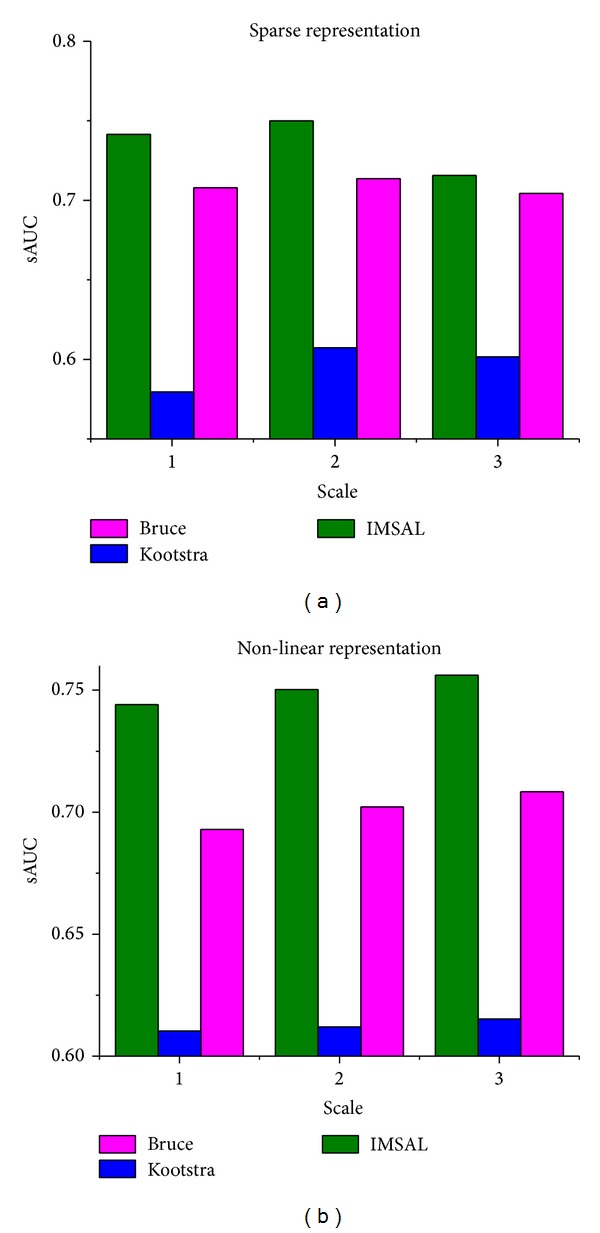
sAUC score for sparse and nonlinear representation. The values in (a) and (b) are maximum at scale 2 and scale 3 for all three datasets.

*Performance Analysis with Resolution*. In order to find the optimal parameters for the proposed model, we treat both representations separately and find the best parameters for each representation that can be incorporated in the proposed model. We plotted both sparse and nonlinear representation with variable resolution on all three datasets and measure the sAUC score. Using various parameters, given in Table 2, Figure 5 is plotted which gives the performance of both representations on the three datasets. Figure 5 shows that, for sparse representation, the performance is maximum with 80 × 60 pixels (scale: 2) and, for nonlinear representation, we get good performance at 512 × 512 pixels (scale: 3). Usually the resolution of the input images is not very high so we restrict to 512 × 512 as upper bound for nonlinear representation and same resolution with respective patch sizes is used in [17]. The image resolution and patch sizes of different scales for the both models used for evaluation are given in Table 2. Based on this analysis, we incorporate the parameters of scale 2 and scale 3 for sparse and nonlinear representation in the proposed saliency model. 

**Figure 6:**
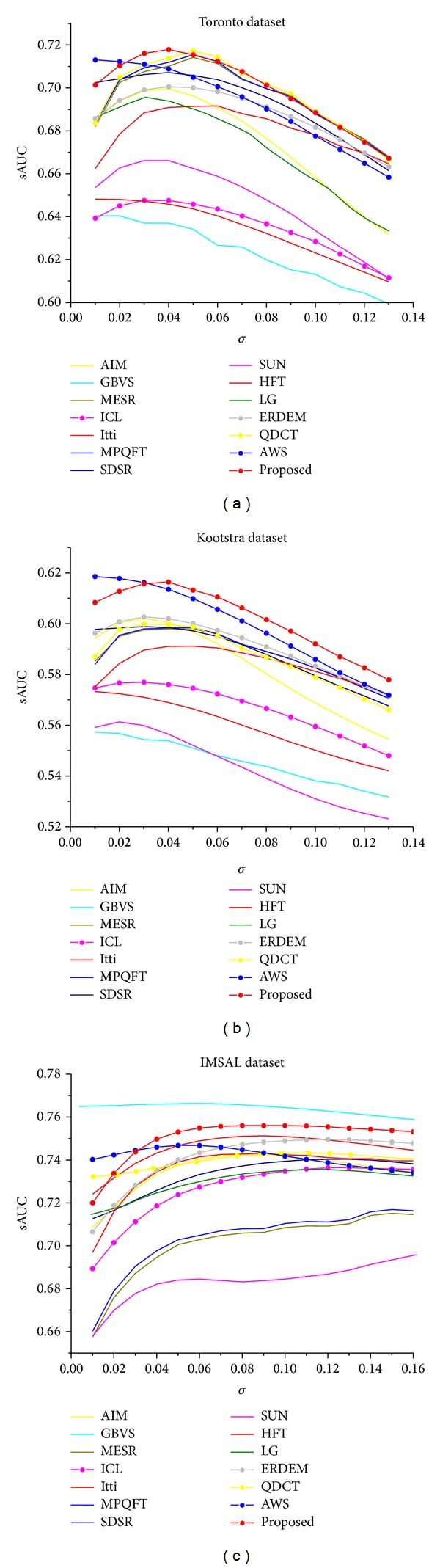
Toronto, Kootstra, and IMSAL datasets with variable Gaussian smoothing for all algorithms in comparison. The *x*-axis represents the *σ* of the smoothing Gaussian (in image width). (In (c) only GBVS, LG, and ΔQDCT are taken from [48]).

*Performance Comparison with Other Models*. The results of our model along with comparison with 13 state-of-the-art methods are given in Table 3. The detailed performance with variable Gaussian smoothing is given in Figure 6. The simulation codes for all these methods are taken from the authors websites. We used multiscale and quaternion based implementation for spectral residue (MESR), PQFT [4], and DCT [49] as proposed by Schauerte and Stiefelhagen [39], which gives higher score than original methods. Erdem's [17] implementation with first order statistics embedded is used for simulation since it gives higher sAUC score. Results with the proposed technique are quite consistent and the proposed method outperforms the state-of-the-art ΔQDCT [39] model on the Toronto dataset and performs comparatively well on the Kootstra dataset and IMSAL dataset. No single model performs well on all these datasets and the performance of other models changes with the dataset but our model shows consistency and remained either ranked 1 or ranked 2 on these datasets. We believe that the high eye fixation prediction is due to the adaptive nature of the model and due to dual representation of features. The adaptivity makes a feature space optimal for the current image; thus, a more accurate representation of the features is possible which in turn accounts for better saliency map estimation. Moreover a single representation may not be enough for every case. Finally, these results can improve further if we use a multiscale representation for ICA based representation, which we skipped due to computational time constraints.

### 4.3. Salient Object Detection

A saliency map can be used to detect a salient object in an image. The basic premise is that if an image consists of an object which stands out from the rest of the image, then it should be identified by a saliency algorithm. There is a different branch in visual saliency modeling which consists of models that are specifically designed to detect salient objects. These models find the salient object in an image and then segment the whole extent of the object and thus solve this task like segmentation-type binary labeling formulation [50, 51]. In contrast, our model is designed for location based (eye fixation) saliency modeling and it is not designed to capture exact object boundaries; however, by thresholding a saliency map, we can get a binary map that can be used for testing performance of a model for salient object detection. Since our saliency model is location based, we only use other location based models in comparison for a fair evaluation, similar convention is followed in [2, 17].

*Dataset and Metric for Evaluation*. For salient object detection, the metric used by Li et al. [2] is area under the curve (AUC) and dice similarity coefficient (DSC) on IMSAL [2] dataset. We will use the same metric and dataset for salient object detection. The DSC gives the overlap between a threshold saliency map and the ground truth. Moreover, the peak value of the DSC [52] is considered an important way to establish the best performance of an algorithm at an optimal threshold; thus, we also give results with peak value of the DSC curve (PoDSC). Since AUC can be influenced by center bias, for fair comparison, we turn off the center bias in all the algorithms. The GBVS [37] has built-in center bias and in order to cater for that, the author in [2] incorporates explicit center bias and shows that HFT [2] performs better than GBVS on the same dataset, which is also used in our paper. We do not employ center bias explicitly or implicitly in the presented results and added HFT instead for our comparison. Therefore GVBS [37] is skipped from Table 4. Furthermore, we perform Gaussian smoothing in all the algorithms to find the optimal smoothing parameters for each class in the dataset and the optimal performance is quoted in the results given in Table 4.

*Performance*. We present results in comparison with other 12 state-of-the-art algorithms. The complete results are given in Table 4. Our proposed scheme gives the best performance on three categories C2, C3, and C5, and ranked second on C4 and C6. Our model gives the highest average AUC score on this dataset. On different categories, our results are comparative to the HFT which is state of the art on this dataset. Apart from HFT, the performance is also significantly better in comparison to other algorithms. The dataset used for comparison is quite complex but our algorithm performed well for intermediate, small objects with distracters although performance on other cases is little less than other state-of-the-art algorithms.

### 4.4. Psychological Patterns

**Figure 7:**
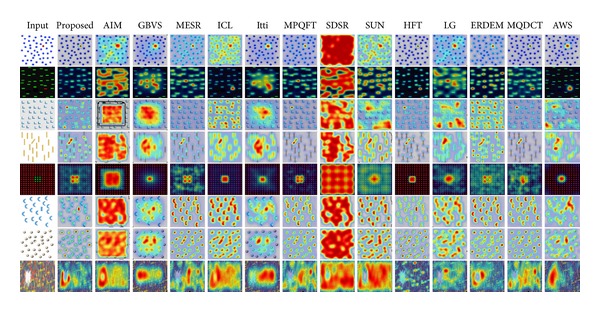
Response of our algorithm and other 13 state-of-the-art algorithms on various psychological patterns.

We also tested our saliency model on psychological patterns, which are commonly used to give a qualitative performance on artificial scenarios which simulate a pop-up phenomenon. These patterns simulate the pop-out phenomenon based on color, intersection, symmetry, orientation, curvature, and candles image. In order to check the general performance on various psychological tasks, we tested the proposed model on eight psychological patterns.  Figure 7 gives the results of the proposed saliency model along with other popular models. The proposed algorithm works well on color, symmetry, and orientation as well as on candle image but the performance is not good for curvature and intersection patterns.  Figure 7 also shows that any single model does not give good performance on all the patterns and the best performance is more or less the same as given by the proposed scheme.

## 5. Discussion

The image features representation drastically affects the information content and thus saliency estimation. We used a bioinspired center surround saliency computation on two parallel feature representations that give good performance on both eye fixation prediction and salient object detection. Since a CSD operation depends on the difference between a center portion and its surroundings, a better image contents representation makes a more accurate and precise center surround operation.

**Figure 8:**
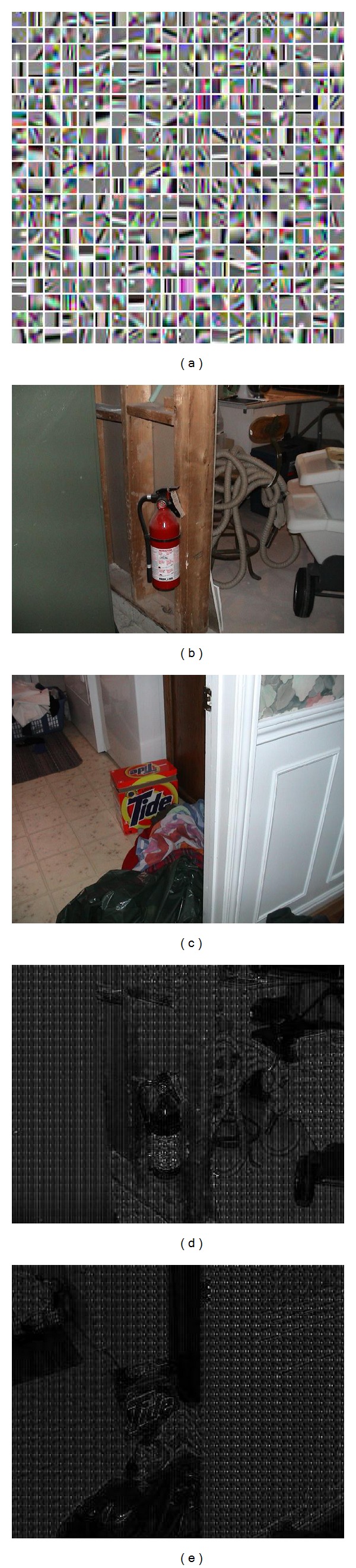
The fixed learned dictionary and the information lost by using such dictionary. (a) Fixed learned dictionary. ((b)-(c)) The input images. ((d)-(e)) The information lost by using dictionary given in (a).

We used an adaptive sparse representation to boost the performance of the CSD operation. In order to show the effectiveness of the proposed approach, we present both quantitative and qualitative results. For qualitative comparison, we use a fix dictionary [29] (Figure 8(a)) learnt from an ensemble of natural images. We show that some of the information is lost if we use a fixed dictionary to represent an image and usually the lost information belongs to the salient region in an image. The difference between an input image and the image reconstructed by a fixed dictionary is given in Figure 8. The red cylinder, Figure 8(b), and red box with text, Figure 8(c), is visible in the image plots Figures 8(d) and 8(e). These two objects are the salient features in both images and their appearance in the residual image shows that current representation, which uses a fixed dictionary, loses some information that belong to salient portion of the input image.

For quantitative comparison, we employ sAUC to do the comparison between saliency maps based on a fixed dictionary and the adaptive basis dictionary.  Table 5 gives a comparison of the both approaches on two datasets, Toronto and Kootstra. The performance difference is quite significant and it shows that an adaptive representation is much better. Based on these qualitative and quantitative results, we can conclude that an adaptive image presentation is more viable and accurate for CSD based saliency computation.

A model proposed in [17] gives an idea of nonlinear integration of all features by covariance matrices and the supported implementation uses color, gradient, and spatial information. Our second contribution is the modification of that model and a proposal of using only color with spatial information in nonlinearly integrated manner using same covariance matrices. For our proposed implementation(see Figure 4), we modified the features used in [17] and compare the saliency map with Erdem's [17] model in Table 6 using the sAUC score. Two databases, Toronto [16] and Kootstra [34], are used for simulations and the results indicate that, by using only color with spatial information, we can get better sAUC score than integrating all features using covariance matrices. In Table 6, the difference in sAUC score is quite visible on both datasets. One possible reason of this improvement may be that the correlation among different features, like color and orientation, is different and thus using a covariance based representation does not capture the underlying information structure in an efficient way as compared to when only color information is used.

One possible argument against our usage of only color information, however, can be that without any gradient or orientation information, a saliency model will fail to detect many salient regions. This argument can also supported by nature since neurons tuned to orientation in an image are known to contribute to saliency computation [53]. In our model, this issue is addressed by the sparse representation where the adaptive basis, same as basis shown in Figure 8(a), is Gabor like filters with edge like structure and thus these bases efficiently capture orientation information from the image which complements our color information in nonlinear representation.

**Figure 9:**
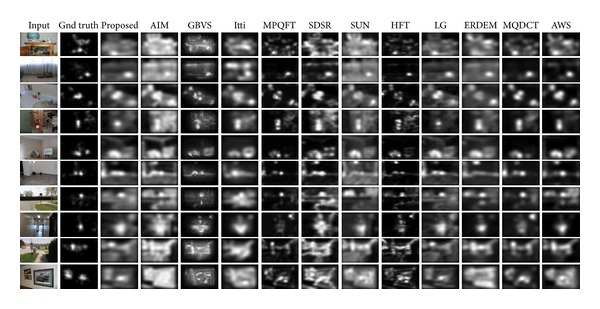
Comparison of our method with other state-of-the-art saliency models. The first column is the input image with second column being ground truth followed by our proposed saliency model. Results from all the other models are plotted in the same row.

Finally, the sAUC score of dual representation, Table 3, shows that we achieve better eye fixation prediction than treating both representations separately as shown in Tables 5 and 6. We believe that such improvement is due to the complementary behavior of both techniques since a combined approach better represents image contents with high fidelity and thus that in turn improves saliency detection. Lastly, for illustration and visual comparison, we present some saliency maps produced by our algorithm along with other models in Figure 9.

## 6. Conclusion

This paper shows that dual feature representation manages to robustly capture image information which can be used in a center surround operation to compute saliency. We show that a CSD on adaptive sparse basis gives better results than a fix sparse basis representation. In nonlinear representation, we show that nonlinearly integrated color channels with spatial information better capture underlying data structure and thus a CSD on such representation gives good results. Finally, we consider both representations as complementary and thus a fused saliency map not only give good results on human eye fixations but also detect salient objects with high accuracy.

In future, we will incorporate some top-down mechanism to better imitative human saliency computation capabilities based on learning and experience. Another possible extension of the existing work is to test dynamic scenes, video, by incorporating additional motion information in the current scheme.

## Figures and Tables

**Table 1:** Following are the input image resolution used in each algorithm with default parameter settings. Some algorithms (at least*) do internally reduce dimensions for fast computation and optimal performance.

Model: Acronym	Resolution
AIM [16]	1/2(*W* × *H*)
*GBVS [37]	*W* × *H *
MESR [39]	64 × 48
*ICL [46]	*W* × *H *
*Itti [37]	*W* × *H *
MPQFT [39]	64 × 48
AWS [23]	*W* × *H *
*SDSR [26]	*W* × *H *
SUN [47]	1/2(*W* × *H*)
HFT [2]	128 × 128
LG [29]	256 × 256
ERDM [17]	512 × 512
ΔQDCT [39]	64 ×48
*Proposed	*W* × *H *

**Table 2:** Various scales parameters which are used for evaluation of sparse and nonlinear representation.

Scale	Adaptive sparse representation		Nonlinear representation	
	Resolution	Patch size	Resolution	Patch size
1	40 × 30	5	128 × 128	{2,4, 8,16,32}
2	80 × 60	5	256 × 256	{4,8, 16,32,64}
3	160 × 120	5	512 × 512	{8,16,32,64,128}

**Table 3:** Comparison of sAUC score of the proposed model with 13 other state-of-the-art models.

Dataset	AIM [16]	GBVS [37]	MESR [39]	ICL [46]	Itti [37]	MPQFT [39]	SDSR [26]	SUN [47]	HFT [2]	LG [29]	ERDM [17]	ΔQDCT [39]	AWS [23]	Proposed
TORONTO [16]	0.699	0.6404	0.7141	0.6476	0.6481	0.7119	0.7062	0.6661	0.6915	0.6939	0.7023	*0.7172 *	0.7130	**0.7179 **
Optimal **σ**	0.04	0.01	0.05	0.03	0.01	0.04	0.03	0.03	0.06	0.04	0.04	0.05	0.01	0.04

KOOTSTRA [34]	0.5901	0.5572	0.5978	0.5769	0.5689	0.5985	0.5984	0.5613	0.5911	0.5938	0.603	0.5998	**0.6185 **	*0.6157 *
Optimal **σ**	0.03	0.01	0.04	0.01	0.04	0.05	0.04	0.02	0.05	0.01	0.03	0.03	0.01	0.03

IMSAL [2]	0.7424	*0.7665	0.7153	0.7364	0.7512	0.71694	0.7403	0.6955	0.7419	*0.7356	0.7455	*0.7434	0.7468	*0.7560 *
Optimal **σ**	0.08	0.07	0.15	0.13	0.09	0.14	0.13	0.16	0.09	0.12	0.12	0.10	0.05	0.09

The top ranked model is in bold font and second ranked model is in italic font. Here results with optimal average sAUC of each method with the corresponding Gaussian blur(*σ*) are reported. As in [47], we repeat the sAUC calculation for 20 times and compute the standard deviation of each average sAUC, which ranges from 1*E* − 4 to 5*E* − 4. (*values taken from [48]).

**Table 4:** Comparison of AUC and PoDSC score of the proposed model with other state-of-the-art models.

Algorithm	C1		C2		C3		C4		C5		C6		Avg.
	AUC	PoDSC	AUC	PoDSC	AUC	PoDSC	AUC	PoDSC	AUC	PoDSC	AUC	PoDSC	Avg. AUC
AIM	0.921	0.653	0.918	0.543	0.957	0.461	0.904	0.435	0.924	0.508	0.941	0.671	0.927
MESR	0.798	0.494	0.854	0.436	0.927	0.377	0.718	0.238	0.837	0.398	0.918	0.617	0.842
ICL	0.928	0.681	0.907	0.501	0.909	0.348	0.926	0.537	0.902	0.498	0.914	0.569	0.914
Itti	**0.939**	*0.687 *	*0.924 *	0.542	0.952	0.457	0.882	0.423	0.924	0.499	0.941	0.631	0.927
MPQFT	0.805	0.497	0.865	0.452	0.927	0.386	0.735	0.248	0.852	0.436	0.921	0.630	0.851
SDSR	0.879	0.618	0.908	0.511	0.948	0.435	0.801	0.298	0.904	0.489	0.933	0.645	0.896
SUN	0.766	0.459	0.787	0.349	0.880	0.362	0.708	0.263	0.719	0.283	0.817	0.451	0.780
HFT	*0.937 *	**0.700**	0.923	0.552	0.937	0.448	**0.964**	**0.648**	*0.933 *	*0.554 *	**0.960**	**0.706**	*0.942 *
LG	0.814	0.515	0.859	0.464	0.904	0.379	0.635	0.145	0.862	0.499	0.884	0.572	0.826
ERDEM	0.882	0.617	0.912	0.555	*0.962 *	*0.526 *	0.840	0.349	0.923	**0.575**	0.916	0.635	0.906
MQDCT	0.840	0.564	0.894	0.517	0.947	0.489	0.806	0.311	0.888	0.524	0.923	0.670	0.883
AWS	0.894	0.625	0.922	*0.569 *	0.957	0.480	0.891	0.402	0.920	0.534	0.925	0.655	0.918
Proposed	0.920	0.653	**0.943**	**0.596**	**0.976**	**0.555**	*0.934 *	*0.516 *	**0.942**	0.532	*0.956 *	*0.688 *	**0.945**

The top ranked model is in bold font and the 2nd ranked model is in italic font. The overall best average AUC score is given by our proposed model.

**Table 5:** sAUC score comparison using fixed [29] and adaptive sparse representation (proposed) for CSD saliency.

Dataset	Borji local [29]: Fixed dictionary sparse representation	Proposed: Adaptive dictionary sparse representation
Toronto	0.670	0.7110
Kootstra	0.572	0.6061

**Table 6:** sAUC comparison of E. Erdem and A. Erdem [17] and the proposed color based nonlinear integration.

Dataset	E. Erdem and A. Erdem [17]	Proposed: Nonlinear representation
Bruce and Tsotsos [16]	0.7023	0.7084
Kootstra et al. [34]	0.603	0.6152
